# Wolf and the Dagger: A Rare Overlap

**DOI:** 10.7759/cureus.87501

**Published:** 2025-07-08

**Authors:** Manasa Tammana, Sarit S Patnaik, Manoj K Parida, Saumya R Tripathy, Bidyut K Das

**Affiliations:** 1 Clinical Immunology and Rheumatology, Srirama Chandra Bhanja Medical College and Hospital, Cuttack, IND

**Keywords:** bamboo spine, genetics, overlap, spondyloarthritis, systemic lupus erythematosus

## Abstract

Systemic lupus erythematosus (SLE) and spondyloarthritis (SpA) are two distinct rheumatological disorders with different pathophysiology. The coexistence of SLE and SpA is very rare and presents unique diagnostic and therapeutic challenges. We present a case of a 28-year-old male who initially presented with inflammatory back pain and characteristic radiologic features, leading to a diagnosis of axial SpA. Subsequently, he developed malar rashes, discoid lupus erythematosus (DLE), and renal involvement. The anti-nuclear antibody (ANA) test by indirect immunofluorescence (IIF) was positive, with multiple antibody specificities on immunoblot. The overlap of SpA and SLE is rare, and a literature review identified only a few case reports. This case highlights the need for heightened clinical awareness and further research into the overlapping mechanisms of SLE and SpA, including potential genetic and immunological factors.

## Introduction

Systemic lupus erythematosus (SLE) and spondyloarthritis (SpA) are distinct rheumatological disorders. SLE is an autoimmune disease characterized by autoantibody production and systemic involvement, whereas SpA is primarily an autoinflammatory condition involving the axial skeleton. The coexistence of these disorders is exceptionally rare, with the first documented case reported in 1982 [1]. To date, only 13 such cases have been described in the literature. Recognition of such overlap is important, as it may influence clinical management and prognostication. For instance, treatments commonly used in SpA, such as nonsteroidal anti-inflammatory drugs (NSAIDs) or tumor necrosis factor (TNF) inhibitors, may have variable effects or contraindications in patients with concurrent SLE due to the risk of lupus flares or drug-induced complications. Similarly, immunosuppressive therapies used for SLE may not adequately address the axial inflammation characteristic of SpA. This report aims to contribute to the limited body of literature by describing a rare case of concurrent SLE and SpA, highlighting diagnostic considerations, therapeutic implications, and the need for further research into the underlying immunological intersection between these two distinct diseases.

## Case presentation

A 28-year-old male patient initially presented to our department six years ago with complaints of inflammatory back pain over the past five years. Additionally, he reported pain and restricted neck movements for the same duration. He had been using alternative medications for symptomatic relief. There was no history of peripheral joint involvement, preceding gastrointestinal symptoms, dysuria, skin rash, nail changes, redness of eyes, blurred vision, or family history of arthritis. Initial investigations revealed elevated acute phase reactants.

**Figure 1:**
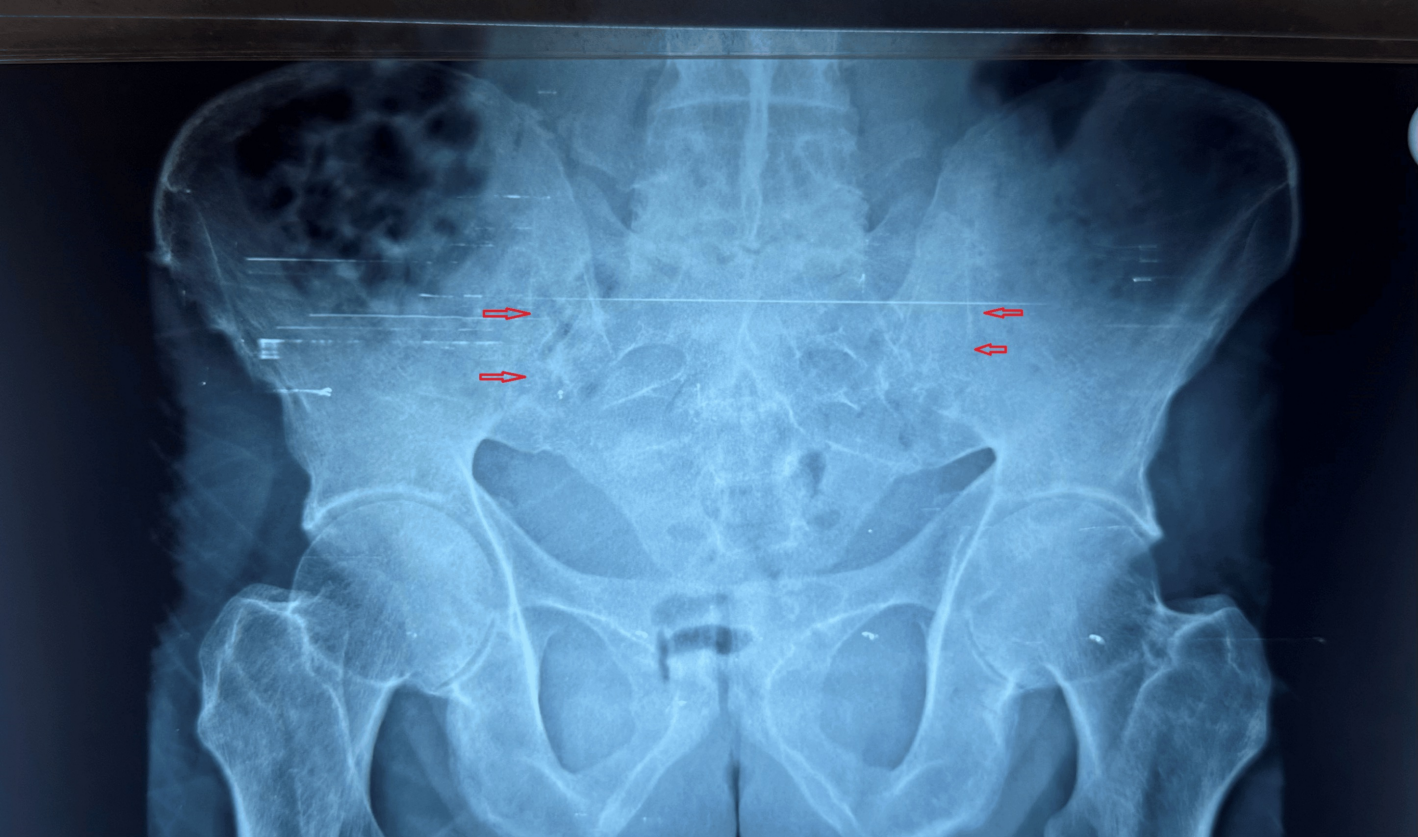
X-ray of the pelvis demonstrating bilateral grade 4 sacroiliitis with joint sclerosis and ankylosis

**Figure 2:**
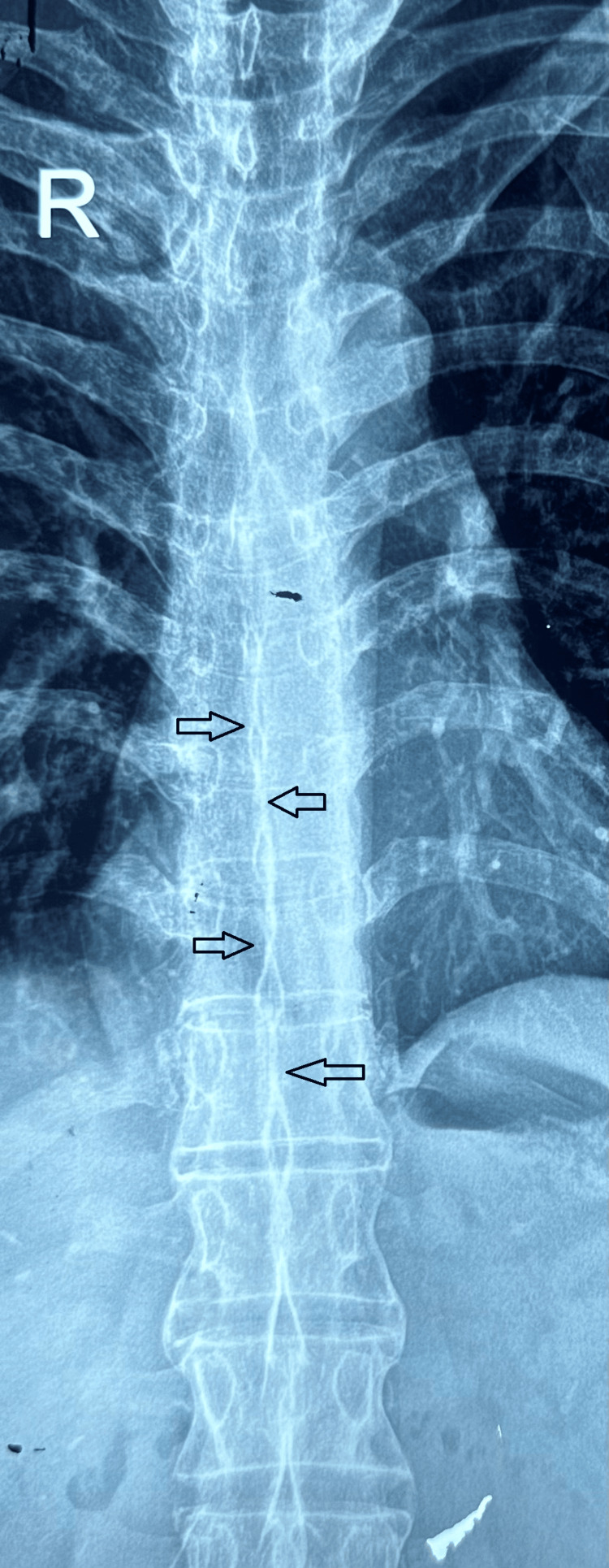
X-ray of the spine revealing syndesmophytes, dagger sign, and a bamboo spine appearance, which is a indicative of advanced ankylosing spondylitis

The human leukocyte antigen (HLA)-B27 by flow cytometry was positive. Radiographic imaging of the pelvis confirmed bilateral grade 4 sacroiliitis (Figure 1), while X-rays of the dorso-lumbar and cervical spine revealed multiple syndesmophytes consistent with bamboo spine (Figure 2). Based on these findings, he was diagnosed with axial SpA and was started on NSAIDs and sulfasalazine, which was gradually titrated to 2 g/day.

However, the patient was irregular with follow-up and had poor compliance with medications. He returned four years later with worsening inflammatory low back pain and neck pain, along with new-onset swelling and pain in bilateral MCP, PIP, ankle, and knee joints. Management included intra-articular steroid injections to the knee and re-initiation of NSAIDs and sulfasalazine.

**Figure 3:**
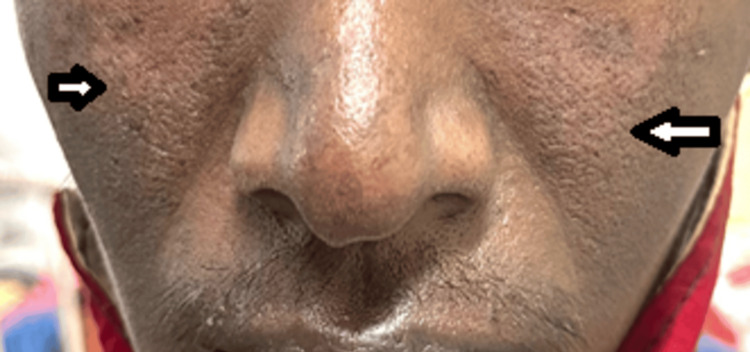
Clinical photograph depicting a prominent malar rash with erythematous distribution across the cheeks and nasal bridge with sparing of nasolabial fold, which is a characteristic of lupus erythematosus

**Figure 4:**
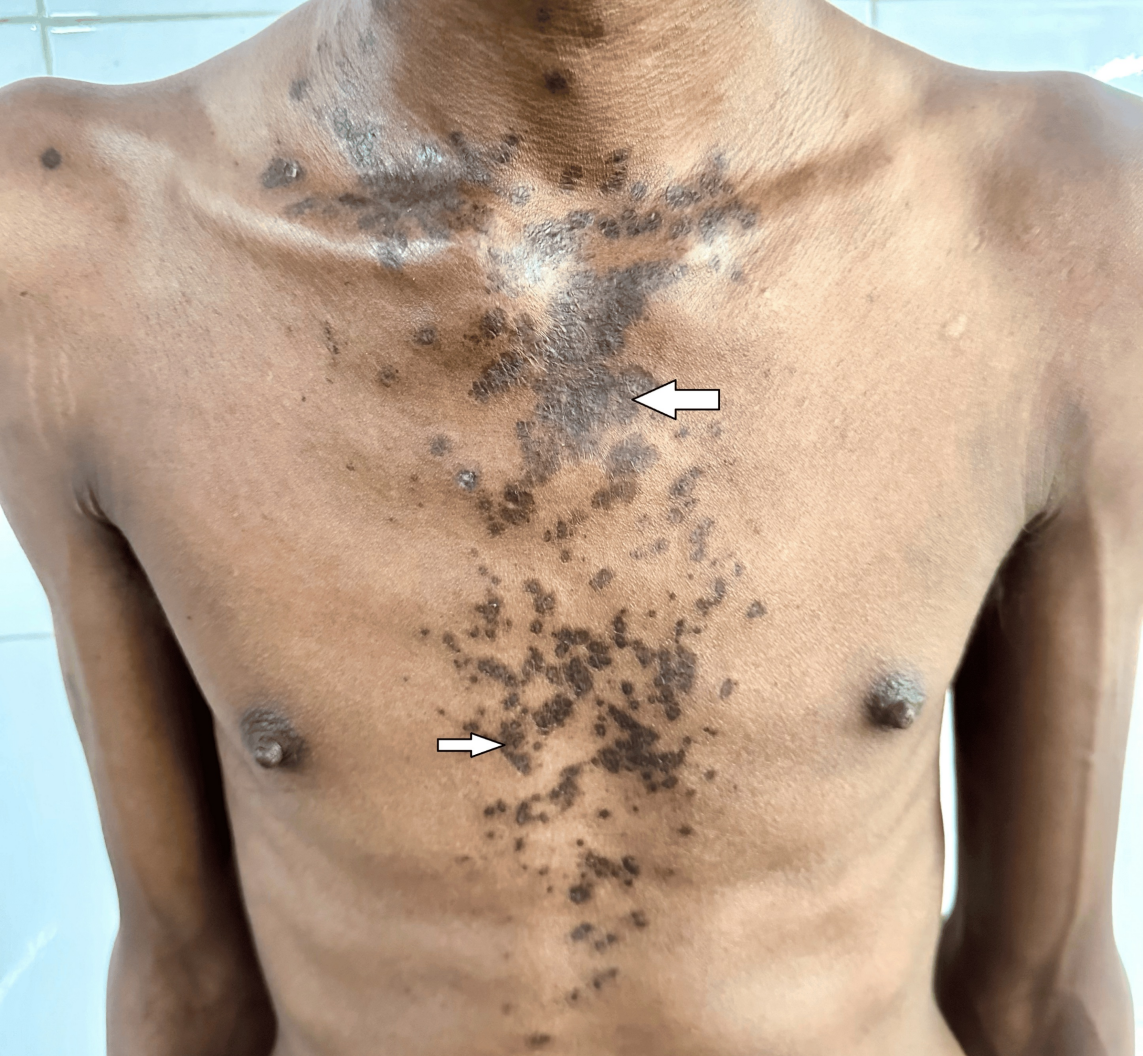
Clinical Image showing multiple well-defined, hyperpigmented discoid lupus erythematosus (DLE) lesions on the chest

**Figure 5:**
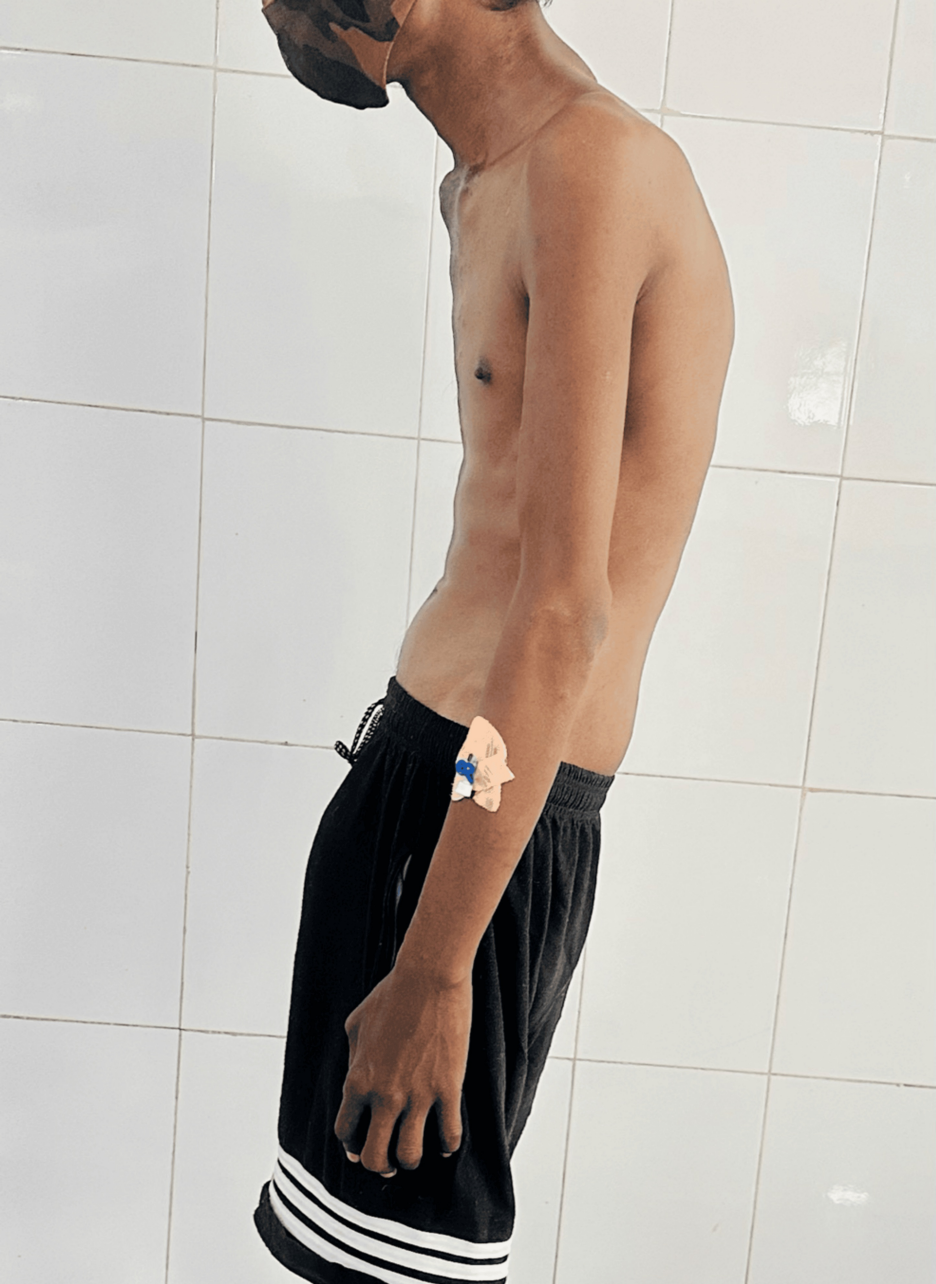
Clinical Image showing increased cervical lordosis and exaggerated thoracic kyphosis, suggestive of structural spinal deformities

In February 2024, the patient presented with new hyperpigmented skin rashes on the chest and triphasic skin color changes of the fingertips on exposure to cold, accompanied by digital pits. He reported no fever, palatal ulcers, pedal edema, facial puffiness, oliguria, or skin tightening. Examination revealed malar rash (Figure 3), multiple annular hyperpigmented maculopapular lesions on the chest (Figure 4), neck, behind the ears, and scalp, suggestive of discoid lupus erythematosus (DLE). The musculoskeletal examination showed boutonnière deformity of the right third finger, increased cervical lordosis, and thoracic kyphosis (Figure 5). Measurements included a wall-to-tragus distance of 26 cm, chest expansion of 2 cm, modified Schober’s of 2 cm, and Bath Ankylosing Spondylitis Metrology Index (BASMI) score of 6.1.

Laboratory evaluation revealed normocytic normochromic anemia, normal leukocyte and platelet counts, and liver and renal function tests. Acute phase reactants were elevated with C-reactive protein (CRP) at 16 mg/L (normal value, <6 mg/L) and erythrocyte sedimentation rate (ESR) at 50 mm/h (normal value, <15 mm/h). Urinalysis revealed proteinuria with no active sediments; 24-hour urinary protein was 884 mg per day. Ultrasonography showed normal-sized kidneys with increased echogenicity bilaterally. The patient was scheduled for a renal biopsy; however, he did not consent to the same. Anti-nuclear antibody (ANA) by indirect immunofluorescence (IIF) showed a 3+ speckled pattern with immunoblot positive for multiple antibodies, including RNP, Sm, and Scl-70. Complement levels were low [C3-24 mg/dL (normal value, >70); C4-13 mg/dL (normal value, >10)], with anti-ds DNA of <10 U/L. A workup for anti-phospholipid antibodies was negative. The Coombs test was negative.

The possibility of sulfasalazine-induced SLE was considered; however, the presence of persistent cutaneous and constitutional disease activity despite stopping the drug for more than six months and the presence of sub-nephrotic range proteinuria made the diagnosis of drug-induced lupus less likely.

The patient was diagnosed with SpA in conjunction with SLE overlap, manifesting as cutaneous Raynaud’s phenomenon with digital pits, arthritis, sub-nephrotic range proteinuria, and presence of multiple autoantibodies with low complements. He was started on prednisolone 0.5 mg/kg, hydroxychloroquine 5 mg/kg, and angiotensin receptor blockers for the same. On the follow-up after three months, the patient had persistent skin lesions with a new onset of erythematous rash over the upper back and active peripheral arthritis, but his 24-hour urinary protein reduced to 397 mg/day. Hence, he was started on methotrexate as a DMARD. After six months of follow-up, the patient is doing well on low-dose steroids, hydroxychloroquine, and methotrexate 25 mg per week.

## Discussion

Search strategy

To conduct a thorough case-based review, a systematic literature search was performed in the PubMed and PubMed Central databases from 1980 to June 2024. The search strategy utilized a combination of keywords and MeSH terms to capture a broad range of relevant articles. The search string included: (“Lupus Erythematosus, Systemic”[Mesh] OR “Systemic Lupus Erythematosus” OR “SLE” OR “Lupus”) AND (“Spondyloarthritis”[Mesh] OR “Spondyloarthritis” OR “Ankylosing Spondylitis”[Mesh] OR “Ankylosing Spondylitis” OR “Axial Spondyloarthritis” OR “Radiographic Spondyloarthritis”). Anti-TNF-induced lupus cases were excluded. Despite this comprehensive search, to date, only 13 case reports detailing the co-existence of these conditions have been identified. These case reports were reviewed and enumerated to assess their relevance and contribute to the overall analysis (Table 1).

**Table 1:** Characteristics of cases with SLE and SpA overlap Anti-dsDNA, anti-double stranded DNA; anti-Sm, anti-Smith; DLE, discoid lupus erythematosus; HLA, human leukocyte antigen; SLE, systemic lupus erythematosus; SpA, spondyloarthritis

S.N0	Cases	Age (years)	Sex	SLE-related symptoms	Anti-nuclear antibody	Other positive lupus antibodies	HLA-B27	Radiography of sacroiliac joint
1	Nashel et al. [1]	43	Male	Malar rash, alopecia, discoid rash, and renal involvement	Homogeneous pattern	NA	+	Grade 4 bilateral sacroiliitis on X-ray
2	Olivieri et al. [2]	42	Female	Malar rash, oral ulcers, alopecia, Raynaud’s phenomenon, digital vasculitis, and renal involvement	Diffuse 1:640	Anti-dsDNA	-	Grade 3 bilateral sacroiliitis on X-ray
3	Korkmaz [3]	26	Female	Arthritis, renal involvement, leukopenia, and thrombocytopenia	Diffuse 1/640	Anti-dsDNA	+	Sacroiliitis on X-ray
4	Chandrasekhara et al. [4]	21	Female	Malar rash, alopecia, Raynaud’s phenomenon, photosensitivity, and anemia	Diffuse 1/640	Anti-dsDNA	-	Grade 2 bilateral sacroiliitis on X-ray
5	Singh et al. [5]	35	Male	Malar rash, discoid rash, renal involvement, and anemia	Diffuse 1/640	Anti-dsDNA	+	Grade 1 bilateral sacroiliitis on X-ray
6	Jiang et al. [6]	29	Male	Malar rash, renal involvement, and anemia	Diffuse 1/640	Anti-dsDNA and anti-SSA	+	Bilateral sacroiliitis on X-ray
7	Mrabet et al. [7]	34	Female	Malar rash, discoid rash, mouth ulcers, and anemia	Diffuse 1/640	Anti-dsDNA	+	Grade 2 bilateral sacroiliitis on X-ray
8	Kook et al. [8]	21	Female	Malar rash and leukopenia	Diffuse 1/640	Anti-dsDNA	-	Bilateral sacroiliitis on X-ray
9	Tarhan et al. [9]	55	Female	Malar rash, renal involvement, anemia, and leukopenia	Diffuse 1/640	Anti-dsDNA	+	Grade 2 bilateral sacroiliitis on X-ray
10	Tanatar et al. [10]	16	Female	Fever, rash, fatigue, oral ulcers, and pancytopenia	Homogenous 1:640	Anti-dsDNA	-	Right-sided active sacroiliitis on MRI
11	Bhavya [11]	64	Female	Oral ulcers and nonscarring patchy alopecia	Homogenous 1:320	Anti-dsDNA and anti-nucleosome	+	X-ray: normal; MRI: not done
12	Bhavya [11]	37	Male	Oral ulcer and alopecia	Homogenous-4+, 1:2560	Anti-dsDNA and anti-Sm	+	MRI: bilateral sacroiliitis
13	DeBoisblanc et al. [12]	50	Male	DLE and renal involvement	Homogenous 1:1280	Anti-histone	-	MRI: partial fusion of left SI joint; subcortical T2 hyperintensity: right iliac bone
14	Current case	33	Male	DLE, Raynaud's phenomenon, and renal involvement	Speckled 3 + 1/100	Anti-Sm, anti-RNP, and anti-Scl-70	+	Bilateral grade 4 sacroiliitis; syndesmophytes on X-ray

The coexistence of SLE and ankylosing spondylitis (AS) is exceedingly rare, with only 13 reported cases worldwide, including the present case. Our patient exhibited definitive clinical and serological evidence of both conditions: SpA, characterized by axial-predominant symptoms, classical radiographic changes, and HLA-B27 positivity; and SLE, evidenced by a characteristic malar rash, Raynaud’s phenomenon, sub-nephrotic range proteinuria, autoantibody positivity, and reduced complement levels.

The relationship between SpA and SLE is particularly intriguing, given their distinct pathogenetic mechanisms. SpA occupies an intermediate space between autoinflammatory and autoimmune disorders, whereas SLE is a quintessential autoantibody-mediated disease. The predominance of SpA in males and SLE in females is well-documented in the literature. Our analysis of the 13 previously reported cases reveals that six were males and seven were females. Interestingly, our case also supports prior findings that the majority of such patients are adults, with only one case reported in the juvenile age group [10].

When comparing the sequence of disease onset, seven of the previously reported cases first presented with AS manifestations, whereas six cases exhibited initial symptoms of SLE. Our patient followed a similar trajectory, with AS manifestations occurring earlier than SLE manifestations. However, in contrast to the homogenous pattern on immunofluorescence reported in other cases, our patient exhibited a speckled pattern, suggesting potential heterogeneity in the immunological response.

Several studies have attempted to elucidate the underlying mechanisms of this rare overlap. For instance, miRNA-16, a small non-coding RNA molecule implicated in autoimmune disease development, has been found to be downregulated in patients with both SLE and AS. This finding supports the hypothesis that common genetic or epigenetic factors may predispose individuals to both conditions [13]. Additionally, while the IL-17/23 axis is well established in SpA pathogenesis, recent studies indicate elevated IL-17 levels in SLE as well. IL-17 is known to directly stimulate B cells to produce autoantibodies, thereby contributing to autoimmune disease progression [14,15]. These findings suggest a potential immunopathogenic link between the two diseases, meriting further investigation.

Despite these emerging insights, the precise mechanisms governing the coexistence of SLE and AS remain poorly understood. The rarity of such cases presents a unique opportunity to explore genetic and immune factors contributing to the simultaneous occurrence of these distinct rheumatological conditions. Furthermore, the overlapping clinical manifestations and potential drug interactions complicate the selection of appropriate immunosuppressive therapy, emphasizing the need for individualized treatment strategies. Future studies are warranted to better understand this overlap and optimize therapeutic approaches for affected patients.

## Conclusions

The coexistence of SLE and SpA, though rare, highlights the complex interplay of immune mechanisms underlying rheumatological disorders. Early recognition of overlapping features is crucial for accurate diagnosis and effective management. Further research is needed to elucidate the genetic and immunological factors contributing to this unique overlap and to guide therapeutic strategies.
